# Spatial–temporal evolution and associated factors of older adult care institutions in Shanghai

**DOI:** 10.3389/fpubh.2025.1598394

**Published:** 2025-07-03

**Authors:** Xingxing Yin, Jinghang Cui, Yifan Wu, Mingxuan Cui, Kun Li, Haoxiang Guo

**Affiliations:** ^1^School of Social Development, East China Normal University, Shanghai, China; ^2^Center for Applied Science in Health and Aging, Western Kentucky University, Bowling Green, KY, United States; ^3^Department of Economics, University of Wisconsin-Madison, Madison, WI, United States; ^4^Department of Cognitive Studies, Vanderbilt University, Nashville, TN, United States; ^5^Shanxi Provincial Water Conservancy Development Center, Taiyuan, Shanxi, China; ^6^Department of Computer Science, University of Rochester, Rochester, NY, United States

**Keywords:** older adult care institutions, spatial distribution, geographically weighted regression (GWR), Shanghai urbanization, aging population

## Abstract

**Background:**

Shanghai is one of the first Chinese cities to tackle the challenges presented by an aging population. In response, the city has been actively seeking solutions for older adult care within a metropolitan context. This study is based on data from 1,272 older adult care institutions in Shanghai. It uses spatial analysis methods to visually display the spatial evolution of older adult care institutions in the city and analyzes the influencing factors of their spatial distribution patterns using a geographic detector approach.

**Methods:**

The research methodology used in the study includes Kernel Density Estimation for visualizing the spatial distribution of older adult care institutions in Shanghai, the Rand Index for measuring the match between older adult care institutions and the older adult population, and Geographically Weighted Regression for addressing spatial heterogeneity by providing local estimates for regression coefficients at different geographical locations.

**Results:**

The number of older adult care institutions in Shanghai has seen significant growth over the past 20 years, increasing fourfold due to the rise in both public and private facilities. Older adult care institutions in Shanghai exhibit clear spatial clustering features, evolving from a central cluster to a pattern of one major center surrounded by multiple secondary centers. The distribution of older adult care institutions and the aging population shows a positive trend, with the matching degree constantly increasing. The mean deviation index (M) decreased from 0.015 in 2005 to 0.011 in 2020. However, resources for older adult care institutions in central urban areas remain relatively scarce, particularly in Xuhui, Jing’an, and Putuo districts. Factors influencing the distribution of older adult care institutions show spatial heterogeneity, with varying correlations with residential, recreational, commercial, transportation-related, and healthcare facilities.

**Conclusion:**

The findings suggest that Shanghai has made progress in expanding older adult care resources to meet the needs of its aging population. However, there are still disparities in the distribution of these facilities, particularly in central urban areas. Understanding the spatial patterns and factors influencing the location of older adult care institutions can guide future planning efforts to ensure equitable access to care for the older adults in Shanghai.

## 1 Introduction

Shanghai was the first city to experience the onset of an aging population in China. As of 2020, the recorded population of individuals aged 60 and above in Shanghai stood at 5.335 million, making up 36.1% of the total registered population. Consequently, Shanghai will be one of the most rapidly aging megacities globally (1). As the population continues to age and the socioeconomic structure evolves, household living arrangements have also undergone notable shifts, with an increasing number of older adults’ individuals choosing to live apart from their children. By 2020, Shanghai was home to 305,200 older adults living alone and 1,577,900 “elder-only” households. Looking ahead, these figures are expected to rise further as the aging trend intensifies. This will place greater demands on the social care service system, community support mechanisms, and family-based older adult care models. It will also prompt both government and society at large to pay closer attention to the quality of life and needs of older adults, and to actively explore more effective solutions for older adult care in order to meet the challenges posed by this demographic shift.

As the city with the highest proportion of older adults in China, Shanghai has taken the lead in exploring institutional older adult care practices. In 2001, the Shanghai government explicitly proposed to establish an institutional older adult care service network covering the entire city. After 20 years of practical exploration, Shanghai has proposed to improve the long-term care service system for the aging population in line with urban development, setting a goal to promote a more balanced and high-quality institutional older adult care service.

The study of institutional older adult care is closely intertwined with the development of population aging (2). Relevant research mainly revolves around the policies of public resource allocation, associated factors, and their effects. Teitz (3) used neoclassical welfare economics to propose the locational issues related to facilities for public interest. Harvey (4) further introduced the concept of spatial justice in public facilities, emphasizing the equitable distribution of resources for public service facilities in different geographical environments to ensure fairness and balance with demand, in order to ensure that different social strata enjoy equal rights. Batta et al. (5) found that from a location science perspective, a reasonable distribution should consider parameters based on population, dispersion, and equity criteria, balancing fairness and efficiency. Schmitt (6) discovered that due to diffusion mechanisms and structural factors, it is difficult to overlook the spatial interdependencies in the distribution of public facilities, and by considering spatial elements solely as exogenous variables, one may neglect the spatial interdependencies in the allocation of public resources in neighboring areas. In comparison, Chinese scholars began their research on institutional older adult care relatively late, but it has developed quickly due to rapid changes in social demographic structures. Presently, the research achievements on institutional older adult care are primarily focused on the following aspects: (1) research on the mismatch between supply and demand in older adult care institutions. Based on life tables, a population actuarial model was established to calculate the age structure of the older adults in Beijing, revealing that the distribution of older adult care beds does not match the spatial distribution of the older adults, leading to the existence of supply–demand contradictions (7). The problem of severe imbalance between supply and demand is attributed to the unreasonable government investment methods, lack of attractiveness of private capital in the institutional care industry, and traditional family caregiving constraints. (2) Efficiency of older adult care institutions. Overall, older adult care institutions in China have low service efficiency, with regional imbalances showing a development pattern of higher development in the east and lower in the west (8). This development pattern is caused by negative effects of cost channels such as wage costs, nursing costs, and relief expenditures on service efficiency, as well as uncertainties in the correlation of policy dividends with service efficiency (9). (3) Challenges in the development of older adult care institutions. Mu (10) proposed a dual dilemma in Chinese institutional older adult care, where there is high demand and low resource utilization efficiency, and poor profitability leading to weak sustainability and self-development capabilities of private older adult care institutions. As population aging continues to progress, the role of older adult care institutions in the older adult care system is becoming increasingly prominent. However, factors such as unclear institutional positioning, lack of detailed service content specification, and a low level of integration of medical and older adult care are placing older adult care institutions in a predicament (11). Additionally, scholars have explored the development of Chinese institutional older adult care from perspectives such as the integration of medical and older adult care (12), government functions (39), and trust crisis (13).

Upon reviewing the domestic and international research progress, it is evident that the issue of older adult care resource allocation has received extensive attention due to the development of an aging population. However, existing studies have largely analyzed the development of older adult care institutions, the living security of older adults, and related welfare systems from a micro-data perspective, overlooking the explanatory power of geographic spatial structure in institutional older adult care development. Research that examines distribution patterns from a spatial–scale standpoint remains scarce. Although some studies have explored the spatial differentiation of older adult care facilities, limitations in data availability, completeness, and continuity have prevented detailed investigations into their spatiotemporal evolution and intrinsic characteristics. As a result, policy targeting is imprecise, social resource allocation is undermined, and optimal configuration and efficient utilization of older adult care resources prove difficult to achieve. To address these gaps, this study employs Python web-scraping techniques to obtain the geographic coordinates of urban older adult care institutions, applies kernel density estimation and matching-coefficient methods to analyze their spatiotemporal differentiation, and utilizes geographically weighted regression to explore the mechanisms underlying older adult care resource distribution. The findings aim to inform urban planning and older adult care policy formulation, promote balanced development of older adult care resources, and offer valuable insights for cities grappling with the challenges of an aging population.

## 2 Data sources and research methods

### 2.1 Research area and data sources

Shanghai is the first city in China to undergo demographic aging and currently holds the highest proportion of senior citizens in the country. The city is divided into 16 districts, including the central urban areas, suburban areas, and outlying districts, classified by Gao and He (14). The central urban areas consist of seven districts: Hongkou, Huangpu, Jing’an, Putuo, Xuhui, Yangpu, and Changning. The suburban areas include four districts: Pudong, Baoshan, Jiading, and Minhang. The outlying districts encompass five districts: Songjiang, Qingpu, Jinshan, Fengxian, and Chongming. There are significant differences in the distribution of older adults in terms of scale and density across districts. As of 2020, Hongkou district records the highest proportion of senior citizens, with those aged 60 or above constituting 42.46% of the total population, while Songjiang district holds the lowest at 29.81%, resulting in a difference of 12.64 percentage points. In terms of density, Huangpu District has the highest density with 1.59 older adults per square kilometer, whereas the Chongming District has the lowest with only 0.02 older adults per square kilometer. The former is 80 times greater than the latter.

The “Shanghai Older adult care Institutions Regulations” issued by the Civil Affairs Bureau of Shanghai define older adult care institutions as facilities that provide centralized residential and care services for older adults, which mainly include nursing homes, homes for older adults, senior apartments, care homes, sanatoriums, retirement care centers, CCRCs, small community organizations, specialized dementia care facilities, and day care centers that offer care beds. Because official channels have not released detailed data on Shanghai’s older adult care institutions, this study employed Python web-scraping techniques to collect location information from high-traffic, reputable platforms such as Yanglao Wang, Yanglao Tiandi, and Lianlao Wang. These websites have long histories in publishing older adult care institution information and therefore offer advantages in temporal continuity, sample completeness, and data accuracy. The datasets gathered encompass a wide range of institutional older adult care types—including nursing homes, senior care centers, convalescent hospitals, older adult communities, and small-scale community facilities—ensuring comprehensive coverage and minimizing errors due to missing samples. To further safeguard data quality and eliminate outliers, anomalous entries were validated against the officially registered corporate credit platforms Qichacha^1^ and Tianyancha^2^. By identifying attributes related to older adult care institutions in Shanghai, including their names, establishment dates, addresses, types of organizations, supply subjects, demand subjects, and service nature, the data was compared with information from the Civil Affairs Bureau and Family Planning Commission to ensure accuracy. This was done through on-site inspections and telephone interviews. Excluding welfare institutions that primarily serve individuals lacking identification papers, a normal residence permit, a source of income, abnormal latitude and longitude data, and null or invalid data, information on 1,272 older adult care institutions was collected. Of these, 634 were publicly funded institutions and 638 were privately owned institutions, with the distribution across different administrative regions shown in Table 1. Based on the geographical locations of these older adult care institutions in Shanghai, they were projected onto the same coordinate system, and the number of older adult care institutions in each district of Shanghai was counted. The data collection period extended until December 31, 2020.

**Table 1:** The number of older adult care institutions in administrative districts of Shanghai in 2020.

Region type	District	Public	Private	Region type	District	Public	Private
Central Urban	Huangpu	73	36	Central Urban	Baoshan	40	61
	Xuhui	29	30		Minhang	35	51
	Changning	32	30		Jiading	33	22
	Jing’an	34	32	Near Suburbs	Jinshan	34	16
	Putuo	38	27		Songjiang	27	16
	Hongkou	25	32		Qingpu	26	20
	Yangpu	40	70		Fengxian	47	26
Near Suburbs	Pudong New Area	76	145		Chongming	45	24

Population data for Shanghai and its respective administrative districts, as well as the numbers of people aged 60 and above and 80 and above, are sourced from the historical editions of “Shanghai Statistical Yearbook” and “Shanghai Older adult care Monitoring Statistics.” When examining the factors influencing the distribution of older adult care institutions, the corresponding POI data were obtained from the AMap Open Platform and classified into five major categories—commercial, medical, recreational, residential, and transportation—based on business-type definitions. The data were subject to verification for authenticity, exclusion of abnormal erroneous data, geospatial coordinate correction, and the establishment of a spatial database through ArcGIS, including the creation of relevant point map layers.

### 2.2 Methods

#### 2.2.1 Kernel density estimation

This study employs Kernel Density Estimation (KDE) method to estimate the spatial clustering degree of older adult care institutions in Shanghai. It identifies the core area of older adult care institutions in Shanghai and effectively describes the characteristics of Points of Interest (POI) spreading from the clustering center to the edges (15). As Equation 1 showing below:


(1) $$p_{n}(x_{i})=\frac{\sum_{j=1}^{n}K(\frac{x_{i}-x_{j}}{h_{n}})}{n\cdot h_{n}}$$

where $p_{n}(x_{i})$ is the kernel density function, and n denotes the total number of older adult care institutions, K () is called the kernel function, h > 0 is the bandwidth, and *x_i_-x_j_* represents the distance from estimation point *x_i_* to *x_j_* (16).

#### 2.2.2 Matching coefficient (MC) and deviation index (M)

Matching coefficient is the ratio of the concentration of older adult care institutions to the concentration of older adults. It represents the density of the spatial distribution of older adult care institutions and older adults in a district in Shanghai. In essence, it reflects the level of match between older adult care institutions and older adults. As Equation 2 showing below:


(2) $$\mathrm{MC}=\frac{R_{{\mathrm{eci}}_{i}}}{R_{{\mathrm{pop}}_{i}}}=\frac{{\mathrm{eci}}_{i}/\sum {\mathrm{eci}}_{i}}{{\mathrm{pop}}_{i}/\sum {\mathrm{pop}}_{i}}$$

where *eci_i_* and *pop_i_* represent the number of older adult care institutions and the older adults in region i at a certain period, while ∑*eci_i_* and ∑*pop_i_* refer to the number of older adult care institutions and the older adults in Shanghai. In general, it is believed that the larger the MC value, the higher the clustering level of older adult care institutions in the region. According to the MC value, the city is divided into resource-abundant districts, resource-sufficient districts, and resource-scarce districts. To further capture the alignment between the distribution of older adult care institutions and the overall older adult population at the higher-level administrative units (17), this study constructs a deviation index (M) comparing older adult care institutions to the older adult’s population. As Equation 3 showing below for calculating the index:


(3) $$M=\sqrt{{\frac{\sum \frac{\sqrt{2}}{2}(\frac{{\mathrm{eci}}_{i}}{\sum {\mathrm{eci}}_{i}}-\frac{{\mathrm{pop}}_{i}}{\sum \text{popi}})}{n}}^{2}}$$

where *n* represents the sample quantity. The larger the Mean Deviation Index (M) value, the higher the degree of deviation between older adult care institutions and older adults in terms of overall distribution, indicating a poorer match between the two. Conversely, a smaller M value suggests a better match.

#### 2.2.3 Geographically weighted regression

GWR models can provide local estimates for the coefficient of function variables at each geographical location. By comparing the spatial variation of coefficient estimates, the spatial variability of variable regression coefficients can be accurately understood (18, 37). The study uses a geographic weighted regression model to conduct spatial regression analysis on the influencing factors of the spatial distribution of older adult care institutions in Shanghai. As Equation 4 showing below:


(4) $$y_{i}=\beta_{0}(u_{i},v_{i})+\sum_{i=1}^{k}\beta_{i}(u_{i},v_{i})x_{\mathrm{ik}}+\varepsilon_{i}$$

where *u_i_* and *v_i_* are the geographic coordinates of the i-th sample space unit; βi(ui,vi) is the value of the continuous function *β_i_*(*u,v*) in the i-th sample space unit. βi changes with the variation of location, and each local *β_i_* is used to estimate its adjacent spatial observations (19, 20).

## 3 Results

### 3.1 The temporal and spatial evolution features of older adult care institutions in Shanghai

#### 3.1.1 Temporal evolution features

##### 3.1.1.1 Spatial evolution features

**Figure 1:**
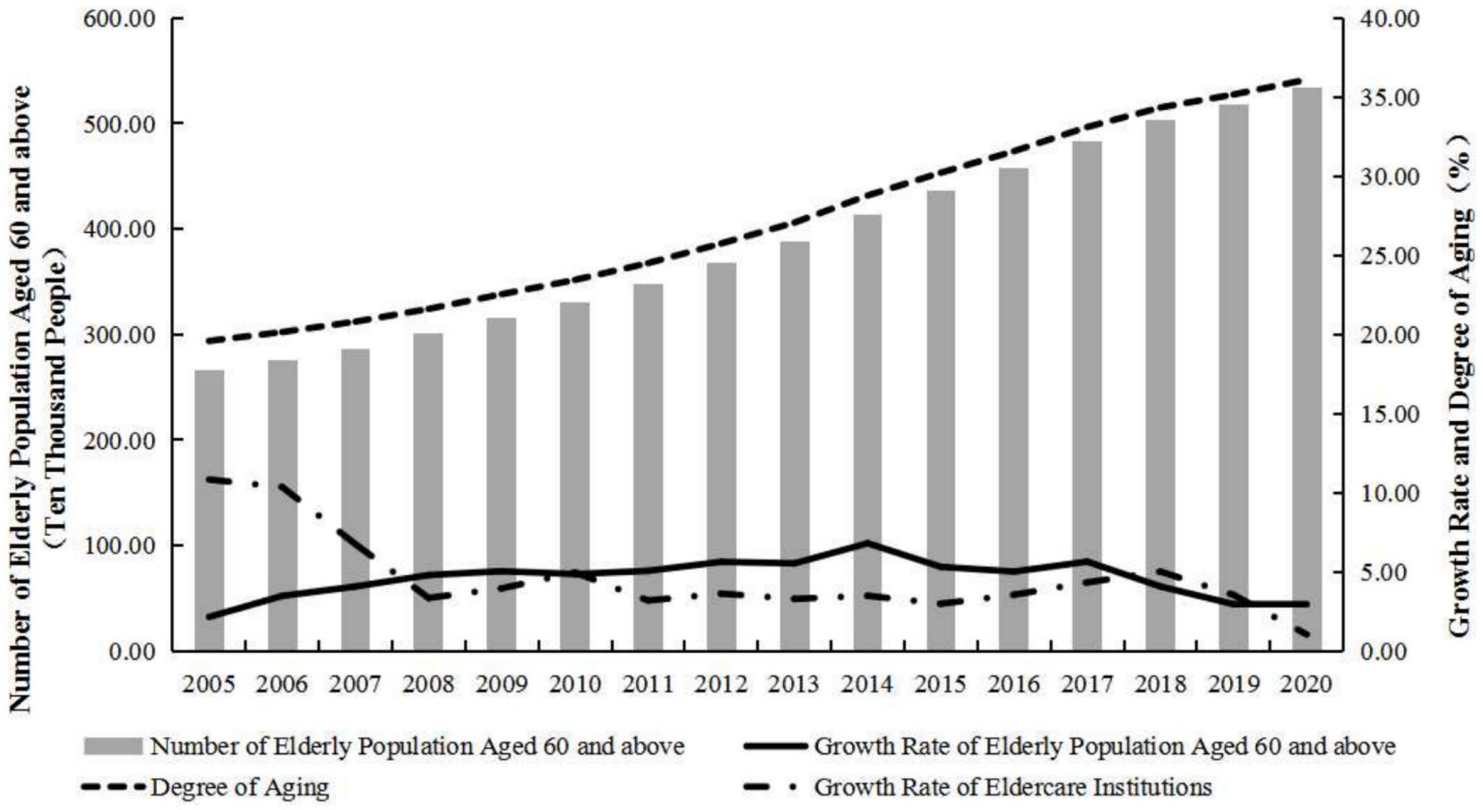
The trends of population aging and the development of older adult care institutions in Shanghai from 2000 to 2020.

The quantity changes of older adult care institutions are closely related to the proportion of older people in the population. During the research period, the proportion of older adults in Shanghai has consistently exceeded the benchmark of 7% for an aging society, showing an overall fluctuating and increasing trend, as depicted in Figure 1. In terms of the changing trend, Shanghai’s aging process has mainly gone through three stages. From 2000 to 2005, it was a period of steady growth in the aging population, with the aging coefficient in Shanghai increasing slowly from 18.31% in 2000 to 19.58% in 2005, with an average annual growth rate of 1.13 percentage points. From 2006 to 2018, it was a period of accelerated aging, with the aging coefficient increasing at an average annual rate of about 4%. In 2019 and 2020, the growth rate slowed down, with an average annual growth rate of 2.5 percentage points. The proportion of older adults in Shanghai increased to 36.09% in 2020, indicating that 1 out of every 3 individuals is an older adult. Furthermore, in 2000, the proportion of older adults aged 80 and above in Shanghai’s population was 2.27%, which grew to 5.58% in 2020, accounting for 15.47% of the total older adult population in the city. It is evident that the aging trend in Shanghai is becoming increasingly severe, and the heavy burden of older adult care will pose numerous challenges to the socio-economic development.

Against the backdrop of continuous population aging and senescence, Shanghai has long been on the path of exploring the concept of “urban older adult care.” During the study period, the number of older adult care institutions in Shanghai showed a rising trend, increasing from 235 in 2000 to 1,272 in 2020, marking a growth of 4.41 times with an average annual growth rate of 8.37%. In terms of institutional features, the rapid growth in the number of older adult care institutions in Shanghai was mainly attributed to the proliferation of private older adult care institutions. Over the study period, the number of public older adult care institutions exhibited a relatively stable growth, with an increase of 477 establishments from 157 in 2000 to 634 in 2020, representing a growth rate of 303.82%. In comparison, the development of private older adult care institutions progressed more swiftly. In 2000, there were merely 78 private older adult care institutions, but over the following two decades, an additional 560 were established, resulting in a total growth of 7.18 times during the study period, with new private older adult care institutions accounting for 54% of the total increase. In terms of development stages, the period from 2000 to 2007 witnessed a rapid growth phase for the older adult care institutions in Shanghai, with an average annual growth rate exceeding 10%. After 2007, Shanghai’s older adult care institutions have maintained a stable growth rate of 3.29% annually.

This rapid expansion from 2000 to 2007 was facilitated by a series of policy implementations: In 2002, the urban planning in Shanghai explicitly stated the necessity to include older adult care institutions in every town; in 2004, the comprehensive promotion of home-based older adult care services was initiated to expedite the development of social older adult care institutions; in 2005, Shanghai pioneered new operational models for older adult care institutions, including the establishment of government-owned private-operated institutions, government-aided private institutions, and the creation of ancillary service areas, actively involving private capital in institutional older adult care services; in 2006, Shanghai expanded the range of medical insurance supplementary fund settlements in older adult care institutions, enhanced the daily operational subsidy mechanisms for older adult care institutions, and vigorously encouraged the participation of non-governmental entities in older adult care services. These initiatives led to a rapid growth in the number of older adult care institutions in Shanghai within a short period of time. Following a phase of rapid growth, the number of older adult care institutions continued to expand, albeit at a slowing rate that tended toward stability. After 2007, as the older adult care market further matures, older adults are no longer content with local older adult care services and have higher expectations for the professional level, business capabilities, and service quality of older adult care institutions. Consequently, the development model of older adult care institutions will gradually shift toward a greater emphasis on quality service.

##### 3.1.1.2 Spatial distribution features

Since the turn of the century, older adult care institutions in Shanghai have experienced rapid growth. In order to better understand the changes in distribution features of older adult care institutions in Shanghai, this study selected three time points, namely 2000, 2010, and 2020, to investigate the spatial differentiation features and distribution pattern changes of older adult care institutions.

The distribution of older adult care institutions has shifted from an even distribution among the central urban area, suburbs, and exurbs to a decreasing trend from the central urban area toward the exurbs. In 2000, there were 81 older adult care institutions in the central urban area, accounting for 34.47%, 78 in the suburbs, accounting for 33.19%, and 76 in the exurbs, accounting for 32.34%. The older adult care institutions were relatively evenly distributed among the central urban area, suburbs, and exurbs. By 2010, the trend has changed to a decrease from the central urban area toward the exurbs. The number of older adult care institutions in the central urban area increased to 369, accounting for 40.50%; 333 in the suburbs, accounting for 36.55%; and 209 in the exurbs, accounting for 22.94%. Compared with 2000, the central urban area and suburbs increased by 6 and 3 percentage points, respectively, while the exurbs decreased by 10 percentage points. By 2020, the number of older adult care institutions in the central urban area increased to 528, accounting for 41.51%; 463 in the suburbs, accounting for 36.40%; and 281 in the exurbs, accounting for 22.09%. The distribution trend of decreasing from the central urban area toward the exurbs has been maintained over the past decade.

**Figure 2:**
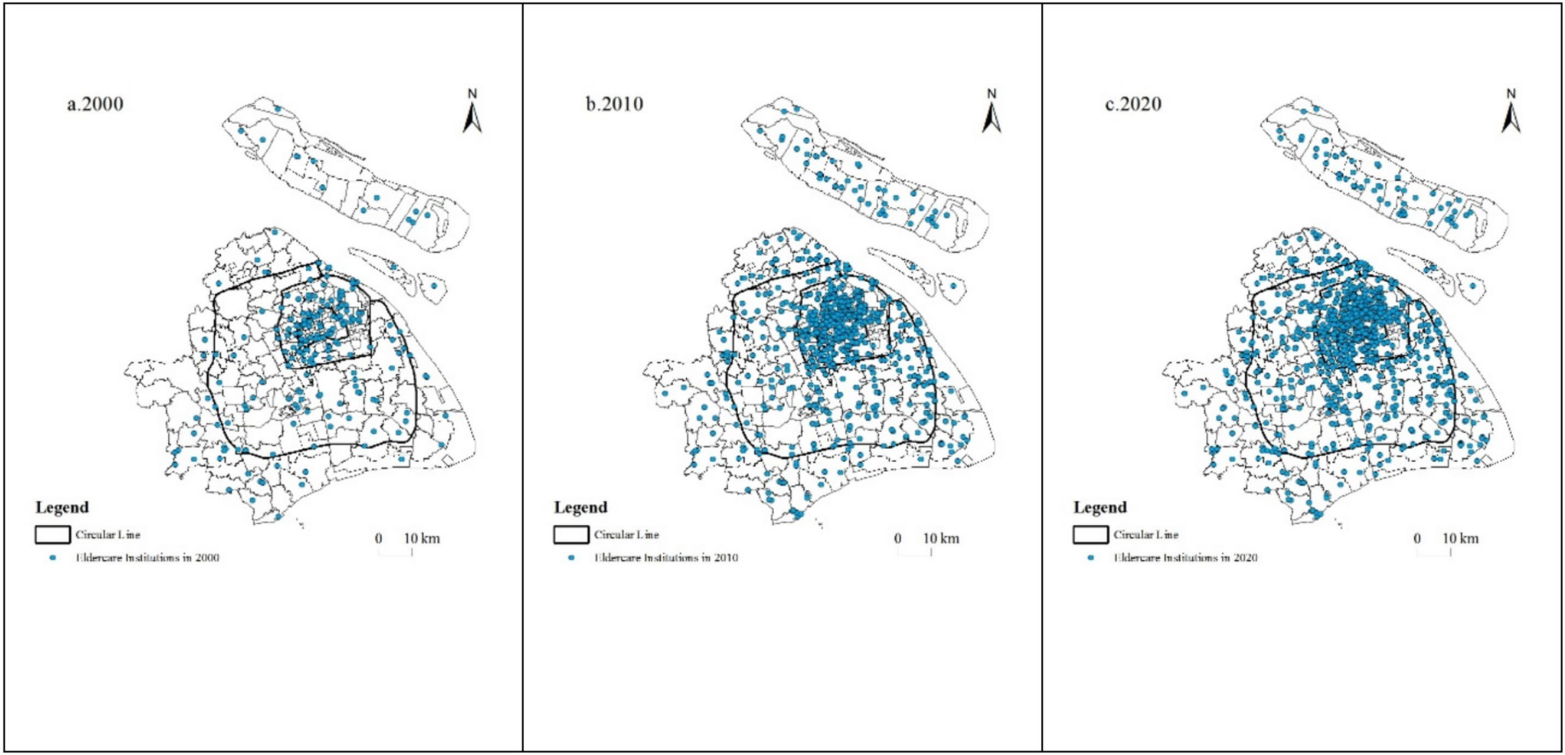
Spatial distribution evolution of older adult care institutions in Shanghai from 2000 to 2020. **(a)** Spatial distribution of older adult care institutions in Shanghai in 2000. **(b)** Spatial distribution of older adult care institutions in Shanghai in 2010. **(c)** Spatial distribution of older adult care institutions in Shanghai in 2020. The black solid lines denote the ring roads, and the blue dots denote the locations of older adult care institutions. From 2000 to 2020, the distribution of older adult care institutions shifted from an even spread across the city center, suburbs, and outer suburbs to a pattern of decreasing density from the city center outward.

The concentration of older adult care institutions within the Ring Expressway became increasingly evident. According to statistical data on the distribution of the Ring Expressway, in 2000, older adult care institutions within the Suburb Ring Expressway accounted for 72.77%, among which 50.21% were located within the Outer Ring Expressway and 18.72% within the Inner Ring Road (Figure 2a). By 2020, the number of older adult care institutions within the Suburb, Outer, and Inner Ring Expressways had increased to 999, 670, and 240, accounting for 78.54, 52.67, and 18.87%, respectively (Figure 2c). The difference in the proportion of older adult care institutions within each Ring Expressway gradually increased, indicating that older adult care institutions were increasingly concentrating toward the inner city despite being located within the suburbs. This suggests that although older adult care institutions in Shanghai are gradually relocating to the exurbs, they remain highly concentrated in the core urban areas.

The growth distribution of different types of older adult care institutions is not synchronized. In the past 20 years, the number of public and private older adult care institutions in Shanghai has increased, with private older adult care institutions growing faster, especially in the exurbs. In 2000, private older adult care institutions accounted for only 33.19% of the total number; by 2010, this proportion had increased to 41.71%, mainly due to the significant growth in the exurbs. From 2000 to 2010, the number of privately operated older adult care institutions in the far suburbs grew by a factor of 7.0—substantially outpacing the 3.4-fold increase recorded in both the central urban area and the near suburbs. Consequently, the far suburbs’ share of the city’s private older adult care institutions rose from 8.97 percent to 14.74 percent. Meanwhile, publicly operated institutions increased by factors of 3.4, 3.1, and 1.2 in the central urban area, near suburbs, and far suburbs, respectively. Thus, although growth rates for public and private institutions were comparable in the central and near suburbs, they diverged significantly in the far suburbs. By 2020, private and public older adult care institutions were evenly divided, with private older adult care institutions growing by 67.89% compared with 2010, higher than the 19.40% growth of public older adult care institutions. In terms of regions, in 2010, the number of public older adult care institutions in the central urban area, suburbs, and exurbs increased by 22.07, 17.95, and 16.99 percentage points, respectively, lower than the increase in private older adult care institutions, which were 74.83, 57.63, and 82.14 percentage points, respectively.

##### 3.1.1.3 The evolution of spatial clustering

**Figure 3:**
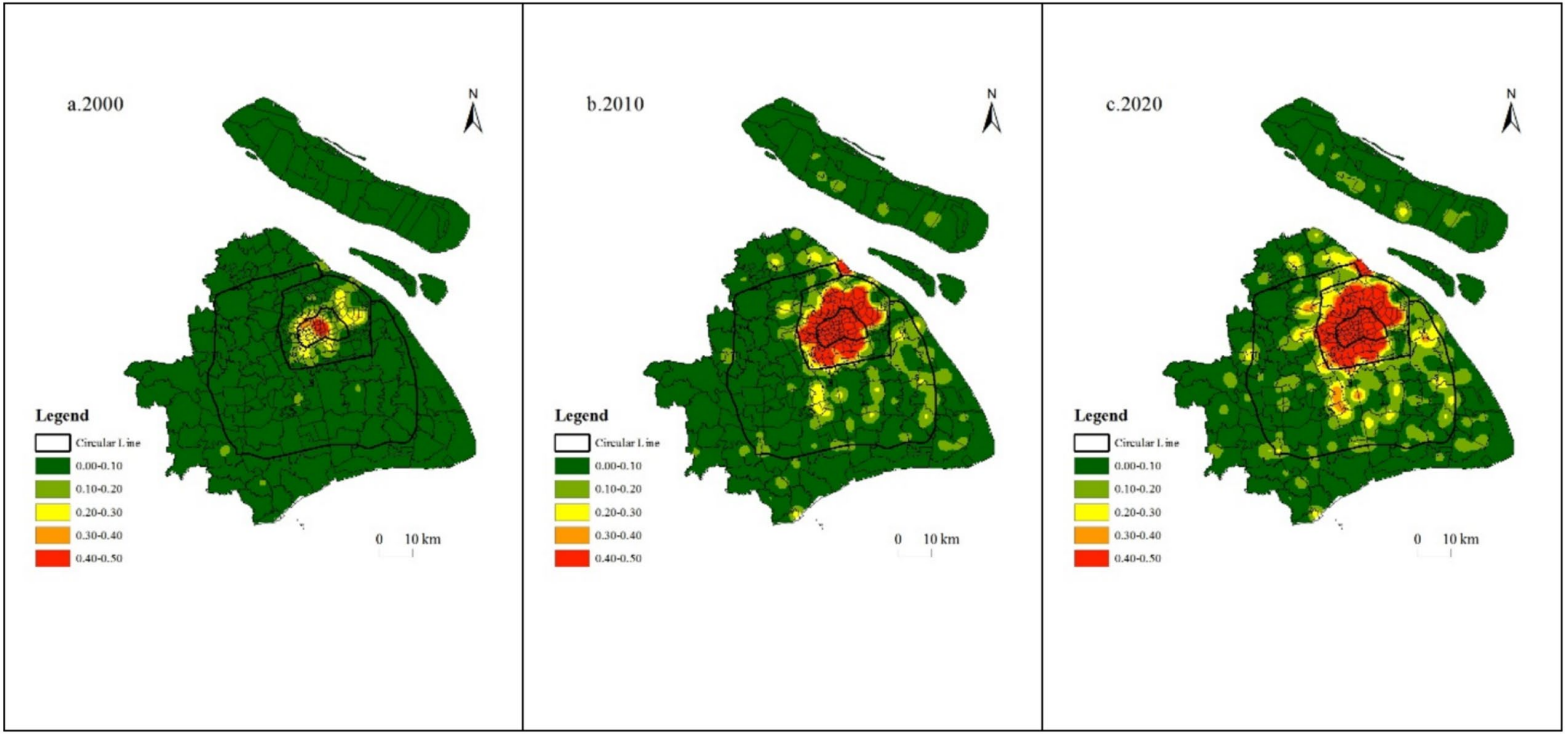
Kernel density analysis map of older adult care institutions in Shanghai from 2000 to 2020. **(a)** Kernel density analysis map of older adult care institutions in Shanghai in 2000. **(b)** Kernel density analysis map of older adult care institutions in Shanghai in 2010. **(c)** Kernel density analysis map of older adult care institutions in Shanghai in 2020. The black solid lines denote the ring roads. From 2000 to 2020, the clustering pattern of older adult care institutions in Shanghai evolved from a “single-center” within the inner ring road to a “one primary and multiple secondary centers” configuration.

To further investigate the clustering features of older adult care institutions in Shanghai, the kernel density estimation method was used to observe the location, extent, and shape features of older adult care institution distribution. During the study period, the spatial clustering features of older adult care institutions in Shanghai were significant, but there were noticeable changes in cluster core, density size, and spatial distribution features, It has gradually evolved from a “single-center” agglomeration within the inner ring road to a “one major center plus multiple subcenters” pattern, in which a primary hub now lies inside the outer ring road while care facilities diffuse in a spot-like fashion across the suburban area beyond it (Figures 2, 3).

In 2000, the area within the Inner Ring Road was a significant region for older adult care institution clustering in Shanghai, centered around Beizhan North Street, Nanjing East Road Street, the Bund Street, Yu Garden Street, Huaihai Middle Road Street, and Laoximen Street, extending outwards to form a high-density circle around the Inner Ring Road, presenting a typical “single center” shape (Figure 3a). According to the distribution density of older adult care institutions, the Huangpu district had the highest distribution density of older adult care institutions with 0.73 older adult care institutions per square kilometer, which is twice the number of older adult care institutions in the Xuhui district (0.31 older adult care institutions per square kilometer), showing a significant disparity.

In 2010, the coverage of high-density areas significantly expanded, reaching from the Inner Ring Road to the inner area of the Outer Ring Expressway, covering almost the entire central urban area (Figure 3b). The density of older adult care institutions in the central urban area increased to 1.27 per square kilometer, showing a substantial improvement compared to 0.27 in 2000. This figure is much higher than the suburbs’ which had 0.14 older adult care institutions per square kilometer and the exurbs’ which had 0.05 older adult care institutions per square kilometer. Huangpu District remains the area with the highest density of older adult care institutions within Shanghai, with 4.06 per square kilometer which is 4.53 times higher than in 2000. Hongkou District, Jing’an District, Yangpu District, and Changning District also had more than 1 older adult care institution per square kilometer, with figures of 1.36, 1.33, 1.23, and 1.12, respectively. Putuo District and Xuhui District had more than 0.5 per square kilometer, while the remaining districts did not exceed 0.2.

By 2020, the central cluster features within the Inner Ring Road remained largely consistent with those in 2010. The number of older adult care institutions outside of the Outer Ring Expressway and within the Suburb Ring Expressway had increased gradually, forming a core aggregation area and a widely scattered diffusion area, exhibiting a distribution of “a large center with multiple sub-centers.” The large center was concentrated within the Outer Ring Expressway; and the surrounding areas of Nanshang, Huacao, Ruiqiao, Jiangchuan Road, Wujing, and Jinqiao Economic and Technological Development Zone, which were outside the Outer Ring Expressway but within the Suburb Ring Expressway, had become the centers of multiple small clusters with relatively lower densities (Figure 3c). The distribution density of older adult care institutions in the central urban area had increased to 1.82 per square kilometer, with the Huangpu district having the highest density at 5.33 per square kilometer. All other six central urban districts had a density exceeding 1 per square kilometer, and all the suburbs had a density exceeding 0.1 per square kilometer. Except for Fengxian district, the number of older adult care institutions in the exurbs was less than 0.1 per square kilometer.

#### 3.1.2 The degree of matching between the aging population and the capacity of older adult care institutions

In order to further investigate the spatial distribution and changes in the matching types of older adult care institutions and older adults in Shanghai, this study calculates the deviation index of each district in Shanghai in the years 2005 and 2020. According to common practice (17, 21–24), this study categorizes the districts into three types based on the degree of matching: resource-abundant districts (MC > 1.2), indicating that the capacity of older adult care institutions exceeds the number of older adults, and the older adult care service system has a certain capacity to accommodate older adults; resource-sufficient districts (0.8 ≤ MC ≤ 1.2), indicating that the capacity of older adult care institutions in the area is roughly equivalent to the number of older adults; resource-scarce districts (MC < 0.8), indicating that the capacity of older adult care institutions is lower than the number of older adults, and further improvement of older adult care services is needed. The matching types and statistical results are shown in Table 2.

**Table 2:** Relationship between the aging population and the number of older adult care institutions in Shanghai from 2005 to 2020.

Year	Category/variable name	Resource-abundant districts (MC > 1.2)	Resource-sufficient districts (0.8 ≤ MC ≤ 1.2)	Resource-scarce districts (MC < 0.8)
2005	Administrative Districts	Huangpu, Jiading, Jinshan, Fengxian, Chongming County	Pudong New, Changning, Putuo, Yangpu, Baoshan, Minhang, Songjiang, Qingpu	Xuhui, Jing’an, Hongkou
	Administrative Districts (%)	31.25	50.00	18.75
	Older Adults (%)	23.97	54.91	21.11
	Older adult care Institutions (%)	32.51	55.69	11.81
2020	Administrative Districts	Huangpu, Fengxian	Pudong New, Changning, Hongkou, Yangpu, Baoshan, Minhang, Jiading, Jinshan, Songjiang, Qingpu, Chongming County	Xuhui, Jing’an, Putuo
	Administrative Districts (%)	12.50	68.75	18.75
	Older Adults (%)	9.61	70.75	19.95
	Older adult care Institutions (%)	14.31	70.43	14.94

At the district level, the consistency between the spatial distribution of older adult care institutions and the aging population in Shanghai has increased, but older adult care institutions in the central urban areas remain insufficient. In 2005, there were eight districts in Shanghai where the distribution of older adults matched with older adult care institutions, accounting for half of the total number of districts in Shanghai. Correspondingly, the number of older adults in these districts accounted for 54.91% of the total number of older adults in Shanghai, and the proportion of older adult care institutions in these districts accounted for 55.69% of the total number of older adult care institutions in the city. At the same time, the other half of the districts in Shanghai were in a state of mismatch between the number of older adult care institutions and the distribution of older adults. Among them, there were five resource-abundant districts, mainly located in the suburbs and exurbs, accounting for 31.25% of the total number of districts in Shanghai. The proportion of older adults in these districts was 23.97%, and the proportion of older adult care institutions was 32.51%. There were also three resource-scarce districts, all located in the central urban areas, accounting for 18.75% of the total number of districts in Shanghai. The proportion of older adults in these districts was 21.11%, and the proportion of older adult care institutions was only 11.81%, accounting for only one-tenth of the total number of older adult care institutions in Shanghai. In 2020, the number of districts in Shanghai with basic matching between older adult care institutions and older adults increased to 11, which is three more than in 2005, accounting for 68.75% of the total districts in Shanghai, exceeding two-thirds. The proportion of older adults in these districts was 70.75%, and the proportion of older adult care institutions was 70.43%. The number of districts with mismatch between older adult care institutions and older adults decreased to five. Among them, there were only two resource-abundant districts, where the proportion of older adults was less than one-tenth (9.61%) and the proportion of older adult care institutions was 14.31%. There were still three resource-scarce districts, all in the central urban areas, where the proportion of older adults was 19.95% and the proportion of older adult care institutions was 14.94%, a difference of five percentage points between the two. In the central urban area, scarce land resources and high land prices impose significant economic pressure on the development of older adult care facilities, driving up construction and operating costs. As a result, many institutions struggle to remain viable or can only offer premium services that are unaffordable for the average older adult resident. Moreover, as the city’s commercial hub, high-demand, high-return commercial development is prioritized in resource allocation, crowding out space for older adult care facilities. With urban plans largely finalized and few opportunities to tap new land resources, the supply of older adult care services is consequently constrained.

Compared to 2005, the number of districts with basic matching between older adult care institutions and older adults increased by three in 2020, accounting for an increase of 18.75 percentage points. The degree of matching between institutional older adult care resources and aging development has slightly increased. The number of resource-ahead districts declined by two, a drop of 12.50 percentage points. Jiading and Jinshan in the near suburbs and Chongming in the far suburbs all shifted from resource-ahead to basic-matching status; their shares of the older adult’s population decreased by 0.78, 0.73, and 1.43 percentage points, respectively. The count of resource-lagging districts remained at three, but their identities changed. In the central urban area, Hongkou District transitioned from resource-lagging to basic-matching: between 2005 and 2020, its older adult’s population share fell by 0.62 percentage points while its share of older adult care institutions rose by 1.42 points, indicating improved alignment. Conversely, Putuo District moved from basic-matching to resource-lagging, as its older adult population share increased by 0.44 points and its institution share declined by 0.62 points, highlighting mounting pressure on service resources.

Upon further examination, from 2005 to 2020, the overall matching between older adult care institutions and older adults in Shanghai exhibited a transformation from a low level to a high level, characterized by a process of “rapid decline — gradual decline — slight increase” in the Mean Deviation Index. Specifically, from 2005 to 2012, the Mean Deviation Index witnessed a rapid descent, decreasing from 0.01478 in 2005 to 0.01103 in 2012, representing a decrease of 25.31%. From 2012 to 2018, the deviation index (M) slowly decreased, reaching 0.01054 in 2018. However, the spatial deviation of older adult care institutions in Shanghai has slightly increased since then, rising to 0.01101 in 2020. In terms of overall trends, the degree of matching between older adult care institutions and older adults in each district has been constantly improving. This promising direction holds great potential for improving older adult care services, optimizing resource utilization across the city, promoting fair access to older adult care services, and achieving equal care standards for older adults overall. It should be noted, however, that even though there is a dense concentration of older adult care institutions in the central urban areas, this does not necessarily mean there is sufficient provision of these services, as the concentration of older adults is also particularly high in these regions. In comparison with the suburbs and exurbs of Shanghai, it is still evident that a higher number of older adult care institutions are required in the central urban areas.

## 4 Analysis of locational factors related to older adult care institutions

### 4.1 Factor selection

Drawing on geographic theory, the siting of older adult care institutions fundamentally reflects a spatial response to multiple interacting factors. Guided by prior studies (25–28), this research selects five categories of influencing variables—commercial, medical, recreational, residential, and transportation—and acquires their corresponding POI data from the AMap Open Platform. Specifically, (1) Commercial, includes supermarkets, shopping centers, and other retail amenities. Because daily necessities such as groceries, banking, and convenience services are indispensable to older adults, the spatial density and accessibility of these commercial facilities directly affect their quality of life and thus influence institutional siting: (2) Medical, Encompasses general and specialized hospitals, community health service centers, nursing stations, and similar healthcare venues. Given older adults’ regular need for medical consultations, medications, and rehabilitation services, the availability and proximity of medical facilities are critical determinants in their choice of older adult care institution; (3) Recreational, Consists of parks, scenic spots, and other leisure spaces. A tranquil, aesthetically pleasing environment not only meets basic recreational needs but also enriches seniors’ cultural and social lives. Areas with ample greenery, clean air, and favorable humanistic environments facilitate outdoor activities, relaxation, and social interaction among older residents; (4) Residential: Refers to housing districts. Older adults typically prefer locations familiar to them and as close as possible to their children’s residences. Moreover, many institutions provide daytime community care, serving seniors who live at home. Hence, the accessibility of surrounding residential areas plays a significant role in institutional site selection; (5) Transportation: Includes airports, railway and bus stations, subway stops, and major road networks. Proximity to highways, rapid transit lines, and public transport hubs ensures timely access to medical and caregiving services, as well as convenient visitation by family members—making transportation accessibility a vital factor for older adults when choosing an older adult care facility.

### 4.2 Analysis results

**Figure 4:**
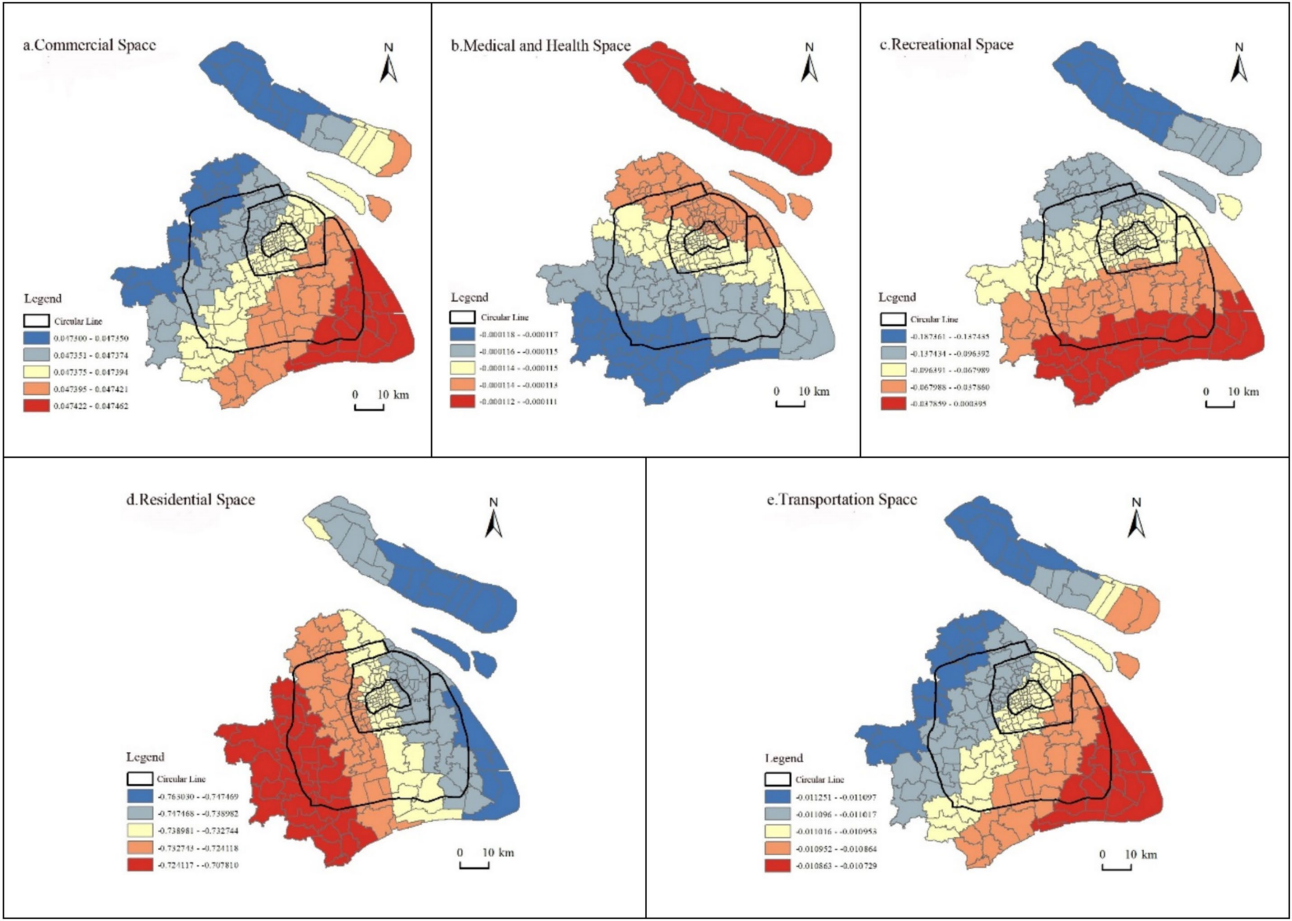
The spatial distribution of regression coefficients for associated factors in the GWR model. This figure displays the spatial distribution of regression coefficients for five factors influencing the spatial distribution of older adult care institutions in Shanghai: commercial space **(a)**, healthcare space **(b)**, recreational space **(c)**, residential space **(d)**, and transportation space **(e)**. **(a)** Commercial space exhibits a positive correlation with the spatial layout of older adult care institutions, with this relationship gradually strengthening from northwest to southeast. **(b)** Healthcare space exhibits a slight negative correlation with the spatial layout of older adult care institutions, with this relationship gradually weakening from southwest to northeast. **(c)** Recreational space exhibits a negative correlation with the spatial layout of older adult care institutions, with this relationship gradually weakening from north to south. **(d)** Residential space exhibits a negative correlation with the spatial layout of older adult care institutions, with this relationship gradually weakening from east to west. **(e)** Transportation space exhibits a negative correlation with the spatial layout of older adult care institutions, with this relationship gradually weakening from northwest to southeast.

Before establishing the model and conducting the analysis, a pre-test should be performed on the data to determine if there is spatial correlation in the distribution of the institutions. If spatial correlation exists, the GWR method will be used for constructing the model, as it provides more accurate parameter estimates. Otherwise, the OLS method should be used. To begin with, the GeoDa software was used to conduct a global spatial autocorrelation analysis on the older adult care institutions in Shanghai. The results show that the Moran’s I values corresponding to the years 2000, 2010, and 2020 are 0.056, 0.068, and 0.094, respectively, passing the test at a significance level of 1%. This indicates that the distribution of older adult care institutions in Shanghai exhibits positive spatial correlation between districts. The development of older adult care institutions in space is not independent, but rather shows a certain degree of clustering features. Additionally, the spatial positive correlation of older adult care institutions gradually increases over time. The spatial distribution of these older adult care institutions does not exhibit complete randomness, but rather shows significant spatial clustering features. Therefore, it does not meet the assumption of spatial independence required by the OLS method. Building the model based on the OLS method would introduce bias. Instead, the GWR model should be adopted to handle spatial heterogeneity. To ensure the reliability of the regression results, we first conducted multicollinearity diagnostics on the five explanatory variables. The variance inflation factor (VIF) for each factor was below 10, indicating that multicollinearity was within acceptable limits. The results of GWR model are shown below (Figure 4).

There is a positive correlation between the distribution of commercial areas and older adult care institutions (Figure 4a). This correlation gradually strengthens from the northwest to the southeast. The correlation between healthcare facilities and older adult care institutions in terms of spatial distribution is relatively low, showing only a weak negative correlation (Figure 4b). There is a negative correlation between the distribution of recreational areas and older adult care institutions. The more recreational areas, such as parks and attractions, there are in an area, the fewer older adult care institutions there tend to be. The distribution of the regression coefficients shows a gradual decrease from north to south (Figure 4c). There is a negative correlation between the spatial distribution of residential areas and older adult care institutions. The denser the residential area, the fewer older adult care institutions there are, and there is a decreasing trend from east to west in terms of spatial distribution (Figure 4d). Transportation has a negative correlation with the spatial location selection of older adult care institutions. The better the traffic conditions, the fewer older adult care institutions there are, exhibiting clear heterogeneity in space, gradually weakening from the northwest to the southeast (Figure 4e).

## 5 Discussion

This study shows that the spatial layout of older adult care institutions in Shanghai is influenced by both policy factors and spatial structure, resulting from their interaction. The distribution of older adult care institutions in Shanghai shows a positive trend with the older population, benefiting from a series of policy implementations. In 2001, the Shanghai government forwarded the “Opinions on Accelerating the Socialization of Social Welfare in Shanghai” proposed by 22 departments including the Civil Affairs Bureau, emphasizing the need to mobilize and rely on social forces to promote the socialization process of social welfare in the long-term and global interests, and to speed up the development of social welfare. The Shanghai government changed its traditional approach of solely constructing and providing services and started to use policy guidance, service purchasing, and financial subsidies to stimulate social investments in older adult care services and encourage the establishment of non-profit older adult care institutions. Meanwhile, the number of publicly funded yet privately operated older adult care institutions has steadily grown. By leveraging market-oriented management and professionalized operations, these institutions have diversified the range of services offered—catering to the varied needs of different senior cohorts—and have markedly improved both the efficiency and quality of service delivery. This model has played a crucial role in lightening the government’s operational burden and optimizing the allocation of resources. By melding the public interest and stability inherent in government support with the flexibility and efficiency of private sector management, it secures both the welfare objectives and operational effectiveness of older adult care facilities. In 2006, Shanghai first proposed a framework for older adult care services based on home care, supported by communities and institutions, and the “9,073” senior-care model which aims for 90 per cent of older adults to be cared for at home, 7 per cent in community care and just 3 per cent in older adult care institutions. Afterward, older adult care institutions entered a peak period of construction (29). With the continuous strengthening of market forces, Shanghai has gradually established a synergistic development model in which the government ensures basic provision while the market promotes diversification, thereby providing robust support for the sustainable development of older adult care services.

From 2012 to 2018, the Mean Deviation Index slowly decreased to 0.01054 in 2018. During this period, older adult care services in Shanghai developed toward a more comprehensive, meticulous, and integrated mode. In 2012, the Shanghai government issued the “Twelfth Five-Year Plan for the Development of Older adult care Services in Shanghai,” proposing to create “age-friendly cities” and “age-friendly communities.” In 2014, in response to the call from the State Council, the “Implementation Opinions of the Shanghai Municipal People’s Government on Accelerating the Development of Older adult care Services and Promoting the Construction of Social Older adult care Service System” was issued, which set a goal to establish a “five-in-one” social older adult care service system for a comprehensive supply, guarantee, policy support, demand assessment, and industry supervision. In the same year, the “Shanghai Older adult care Institutions Regulations” were issued, which required reasonable distribution of older adult care institutions and the formulation of special plans for the distribution of older adult care facilities. The commitment to comprehensive improvement of older adult care service quality has also promoted further matching of older adult care institutions and the aging population. From 2018 to 2020, the Mean Deviation Index slightly increased to 0.01101 in 2020. In 2015, the Ministry of Civil Affairs, the National Development and Reform Commission, and other 10 departments jointly issued the “Implementation Opinions on Encouraging Private Capital to Participate in the Development of Older adult care Services,” which encourages private capital to participate in institutional older adult care services and supports private capital to participate in the development of the older adult care industry. In 2017, the State Council issued the “Notice of the Thirteenth Five-Year Plan for the Development of National Older adult care Services and the Construction of an Older adult care System,” which encouraged chain operations, promotes group development, and builds branding for older adult care institutions, emphasizing the need for older adult care institutions to enhance service capabilities in market competition. The private capital was actively mobilized to participate in the older adult care services, greatly activating the vitality of older adult care institutions, and also raising the Mean Deviation Index.

The “Opinions on Strengthening the Comprehensive Regulation of the Population in Shanghai” in 2002 suggested encouraging older adult care institutions to relocate from expensive central urban areas to the more affordable suburbs and exurbs, where the environment is also better. However, in reality, older adult care institutions continue to expand in the city center despite the formation of several smaller center aggregation areas in the suburbs and exurbs (38). This concentration trend is primarily based on the distribution of the aging population. The city center has well-allocated resources but is densely populated with a higher proportion of older adults. On the other hand, the peripheral areas, positioned as urban functional expansion zones with abundant land resources suitable for establishing larger-scale older adult care institutions, have a lower proportion of older residents. This spatial mismatch is not only detrimental to meeting the institutional older adult care needs of older adults in the city center, but also inhibits the formation of retirement service centers with aggregated resources in the surrounding areas, leading to a “shortage of beds” in the central urban areas and “idle beds” in suburbs.

Spatial inequality is the manifestation of social inequality across geographic dimensions, and spatial justice theory emphasizes fairness in resource allocation, participatory rights, and the use of space. In the context of rapid urban development and spatial differentiation, urban social policy must adopt spatial justice as a guiding principle to ensure equitable distribution of resources. Under the goal of “equalizing basic public services,” planning the spatial distribution of older adult care institutions in large cities should be grounded both in the distribution of the older adult’s population and in the city’s overall spatial framework. By integrating older adult’s population density with the status of basic regional service facilities, resource allocation can achieve precise alignment between older adult care resources and social needs. Moreover, through systematic planning procedures and mechanisms that engage multiple stakeholders, the principles of distributive justice, procedural justice, and participatory justice within spatial justice theory can be realized—ensuring that older adults across all districts and social strata have equal access to older adult care services. In urban areas where there is a high concentration of older adults and a high demand for older adult care resources, but an insufficient supply, it is possible to develop existing resources and renovate facilities in order to increase the availability of older adult care resources. Government departments should expedite the implementation of favorable policies and effectively guide older adults to relocate to regions that offer ample older adult care resources to alleviate the strain on older adult care resource availability in the city center. For regions with low density of the aging population and low pressure on older adult care resources, efforts can focus on strengthening the construction of supporting facilities and enhancing the quality of older adult care services. This can leverage their driving force on surrounding areas, promoting the overall coordination and balanced development of older adult care resources across the entire domain. Furthermore, it is imperative to encourage market forces to play a role in areas where institutional older adult care is lacking, especially considering the flexible and diverse responsiveness of private older adult care institutions. In its early stages, institutional older adult care primarily focused on welfare and public service, with relatively low market participation. However, as the trend of aging population intensifies and the level of social and economic development progresses, the institutional older adult care market has gained momentum. In addition to traditional public and private older adult care institutions, a variety of public-private collaborative supply models have emerged, such as government-owned private-operated institutions and government-aided private institutions. Facilitating the supplement of older adult care resources through market forces can contribute to achieving spatial balance of older adult care institutions.

The organizational and institutional arrangements shape and delineate the spatial layout of older adult care institutions in Shanghai. Nonetheless, these institutions also adapt accordingly based on the current spatial distribution of other social resources. Space is a product of social production and at the same time a reproducer, playing a decisive role in the continuity of social reproduction (30). Henri Lefebvre posits that “social space” is diverse, with broader “social space” encompassing various smaller “social spaces,” known as “social subspaces” (31). Older adult care institutions coexist with commercial, medical, recreational, residential, and transportation spaces, all considered “subspaces” of “social space.” These subspaces interdependently interact with each other, engaging in “self-production” and “mutual production,” nested and combined, forming an intricate, dynamic, and organic social whole that influences the allocation of public welfare resources (31). From the standpoint of spatial justice theory, the allocation of spatial resources must uphold fairness and equity. When subspaces develop in a coordinated manner, spatial justice is realized, enabling a balanced distribution of public welfare resources across different areas and demographic groups. However, if these subspaces become uncoordinated—such as when commercial expansion encroaches on older adult care and recreational spaces—seniors are left without suitable venues for activity, and public welfare resources become skewed toward commercial interests. Such imbalances violate the principles of equal and just resource distribution championed by spatial justice theory and undermine the sustainable provision of public welfare services. The study reveals that the spatial distribution pattern of older adult care institutions in Shanghai is influenced by commercial, medical, residential, recreational, and transportation spaces. Apart from commercial space showing a positive impact, the rest exhibit negative correlations, albeit with significant regional disparities. This implies that a greater presence of large supermarkets and shopping centers is associated with a higher number of older adult care institutions. The distribution density of commercial areas is higher in the Northwest region compared to the Southeast region. There is a lower correlation between commercial areas and older adult care institutions in the Northwest region, while in the Southeast region, the correlation between the two is higher. Due to the relatively limited presence of supermarkets and shopping centers in the Southeast region, older adult care institutions need to be built in nearby areas for the convenience of older adults to purchase essential goods. The correlation of their placement is relatively significant in commercial areas. On the other hand, in the Northwest region where there are more supermarkets and shopping centers, the location selection for older adult care institutions is comparatively more flexible. In the southeast of Shanghai, in Pudong New Area and Fengxian District, new older adult care institutions are being added. It is suggested that during the site selection process, careful consideration should be given to the location of commercial facilities in order to layout the area effectively, and to be as close as possible to shopping centers for the convenience of older adults residents.

Although medical and health institutions exhibit only a slight negative correlation with the spatial distribution of older adult care facilities, notable regional differences persist. In Chongming District—located in the northeast, where hospital resources are relatively scarce and dispersed—the impact of healthcare institutions on older adult care siting is limited. By contrast, in the southwestern districts, the presence of medical facilities plays a more pronounced role in shaping the layout of older adult care institutions. This may be because most older adult care institutions in Shanghai have medical facilities and healthcare personnel, which can meet the daily healthcare needs of older adults living in these institutions. At the same time, the medical conditions and healthcare services provided by older adult care institutions are important factors considered by older adults when choosing to reside in older adult care institutions. The willingness of partially self-reliant and entirely self-reliant older adults to live in older adult care institutions is decreasing, while the willingness of disabled older adults to reside in these institutions is increasing (32). The integration of medical and older adult care services breaks down the traditional barriers between healthcare and senior care by deeply embedding modern medical technologies into older adult care resources, thereby establishing a novel support model of “treatment when ill and preventive care when healthy.” This approach not only meets older adults’ multi-level and diverse health needs and enhances their quality of life and well-being, but also improves the utilization efficiency of both healthcare and older adult care resources, reducing social costs and fiscal burdens (33). Older adult care institutions with strong expertise, high nursing standards, and good medical conditions are preferred and can have access to better medical resources than home-based and community-based care. It is recommended that in the future; efforts be made to actively respond to the development strategy of integrating medical and older adult care. This can be achieved by equipping existing nursing homes with medical facilities to enhance their medical capabilities, catering to the needs of older individuals with advanced age or disabilities. Additionally, policy interventions should be utilized to promote partnerships between medical institutions and nursing homes, thereby improving the quality of services provided.

The distribution density of residential areas in the eastern part of Shanghai is slightly higher than in the western part, indicating a relatively stronger correlation between residential areas and older adult care institutions in the densely populated eastern region compared to the less populated western region. There are three possible explanations. Firstly, in residential areas with relatively high population density, typically located in easily accessible and commercially developed regions, land prices are relatively high. As older adult care institutions are non-profit organizations that serve a certain social welfare purpose, even with government policies and financial support, it is difficult for them to choose locations with high land prices. Secondly, improving living conditions is one of the reasons why older adults choose older adult care institutions. Shanghai has various types of residential areas, including traditional alleyways, high-rise residential areas, rural resettlement areas, and rural housing. According to a recent survey, 82% of older adults in Shanghai reside in houses built before 2000 (34). These neighborhoods are not suitable for older adults due to their small size, lack of older adult care facilities, and inadequate safety measures. Additionally, the presence of non-elevator multi-story residential buildings greatly restricts the activities of older adults with mobility issues. The care of children and the satisfaction of older adults with home-based older adult care are significantly related to their intention to reside in older adult care institutions. Older adults who are less satisfied with the care provided by their children and experience less harmonious family relationships have a higher tendency to choose to live in older adult care institutions. The proximity of the older adults’ residence to their children’s dwelling does not have a positive correlation with their intention to reside in older adult care institutions (35, 36).

The distribution density of parks and attractions in the northern part of Shanghai city is higher than in the southern part. The northern region, with more abundant parks and attractions, has a greater impact on the distribution of older adult care institutions, while the impact is relatively smaller in the southern region with fewer leisure spaces. The areas with more parks and attractions have superior natural environments, pleasant settings, and good cultural environments, making them popular tourist destinations with high levels of human and vehicular traffic flow, which may not be conducive to older adults seeking older adult care services. In contrast, the northwest region adjacent to the inland has more highways and higher levels of transportation development, exerting a greater influence on the spatial layout of older adult care institutions, while the southeast region adjacent to the sea has weaker transportation networks and thus a relatively weaker impact on older adult care facilities. This may be attributed to the fact that areas with well-developed transportation facilities usually have higher human traffic flow and noisy environments, making them less suitable for older adult care services.

Accordingly, future allocation of social public resources should move away from the traditional approach of targeting single resource types in isolation. Instead, attention must be paid to how the various subspaces within a region interact, with targeted layouts and adjustments designed to foster “multi-circle integration” among older adult care institutions, residential areas, medical zones, recreational hubs, commercial centers, and transportation networks. For example, in the central urban area—where land is scarce and population density is high—efforts should focus on developing small-scale, specialized, and finely managed older adult care facilities that collaborate closely with nearby mixed-use commercial complexes and leisure venues to create a one-stop older adult care ecosystem, thereby offering seniors more convenient daily services. In suburban districts, the abundant natural landscapes and tourism resources can be leveraged to establish eco-recreational older adult care models. At the same time, given the relative dispersion of medical resources in these areas, strengthening partnerships between older adult care institutions and medical networks will enable resource sharing and two-way referrals. Moreover, to avoid perpetuating unequal regional interactions, inter-regional cooperation and exchange should be enhanced to promote the sharing and complementation of older adult care resources, achieving coordinated and sustainable distribution of institutional care across regions and providing seniors with higher-quality, more convenient, and more diversified services.

There are three main shortcomings in this study: first, when considering the factors influencing the spatial location of older adult care institutions in Shanghai, the study did not take into account population, economy, institutional factors, etc. These factors could only be accessed at the district level of data. Obtaining data at a smaller scale and incorporating it into the study could further improve the accuracy of the research results. Second, this study considered only the number of older adult care institutions. Owing to data availability constraints, we were unable to examine institution scale, bed capacity, service quality, or occupancy rates. Furthermore, due to limitations in our data-collection methods, unregulated or unregistered facilities were not captured in our analysis. These factors are essential components of older adult care service provision and are critical for a comprehensive evaluation of the older adult care system. Third, we did not investigate demand-side variables such as older adult income stratification (e.g., pension levels), intergenerational living arrangements (living alone versus co-residence with children), or migration patterns (e.g., “migratory” older adult care). This omission leaves gaps in our understanding of the mechanisms driving spatial choice. In future research, we plan to conduct field visits, telephone surveys, and other data-collection activities to gather detailed information on institution scale, bed counts, medical services, fee structures, service provision, occupancy rates, and older adult residents’ needs, thereby extending and deepening the present study.

## 6 Conclusion

Utilizing Python web scraping technology, information on 1,272 older adult care institutions in Shanghai from the years 2000 to 2020 was obtained. Through spatial modeling analysis, this study examined the spatial distribution features of older adult care institutions in Shanghai and their correlation mechanisms. The main conclusions are as follows:

First, during the study period, the number of older adult care institutions in Shanghai showed a growing trend, increasing from 235 in 2000 to 1,272 in 2020, a growth of 4.41 times. This growth was mainly attributed to the stable development of public older adult care institutions and the rapid increase in the number of private older adult care institutions. Over the twenty-year period, public older adult care institutions in Shanghai grew by 3.03 times, while private older adult care institutions grew by 7.18 times. In 2020, public older adult care institutions and private older adult care institutions in Shanghai each accounted for half of the total.

Second, from the spatial distribution perspective, older adult care institutions exhibit a central aggregation in the urban core area and a scattered distribution near the exurbs, with the quantity decreasing from the central urban area toward the exurbs. Although older adult care institutions in the newly developed suburbs and exurbs are gradually increasing, the tendency for older adult care institutions to be concentrated within the Ring Expressway still persists. Despite the increase in older adult care institutions locating in suburbs and exurbs, the growth rate of institutions in the urban area is faster, leading to a widening gap in the ratio of older adult care institutions inside and outside the Ring Expressway, strengthening the trend of concentration toward the inner regions. Overall, during the research period, the distribution of older adult care institutions in Shanghai has undergone a transformation. Previously, the concentration was centered around Beizhan North Street, Nanjing East Road Street, the Bund Street, Yuyuan Street, Huaihai Middle Road Street, and Laoximen Street, forming a “single center” aggregation. It has now shifted toward a “one main center with multiple sub-centers” distribution pattern, with the main center being within the Outer Ring Expressway and the sub-centers scattered within the Suburb Ring Expressway.

Third, in terms of the matching degree between older adult care institutions and the aging population, the distribution of older adult care institutions in Shanghai shows a positive trend toward better matching with the aging population, and the matching degree has been continuously improving, resulting in more equitable urban older adult care services for older adults. Although the distribution of older adult care institutions is becoming increasingly concentrated in central urban areas, the number of older adults in these areas is growing at a faster rate. Therefore, the supply of older adult care resources can only meet the demand to a certain extent.

Fourth, the distribution of older adult care institutions is highly correlated with residential areas, gradually decreasing from east to west; the correlation with recreational areas gradually decreases from north to south, while the correlation with commercial areas gradually increases from northwest to southeast, and correlation with transportation gradually decreases from northwest to southeast. The correlation with healthcare facilities is the lowest, gradually decreasing from southwest to northeast. Therefore, it is important to consider the regional and specific differences in the correlation of various factors when planning the layout of older adult care institutions, in a rational and scientific manner.

## Data Availability

The original contributions presented in the study are included in the article/Supplementary material, further inquiries can be directed to the corresponding author.

## Footnotes

^1^ https://www.qcc.com/

^2^ https://www.tianyancha.com/
